# Wearing Face Masks Strongly Confuses Counterparts in Reading Emotions

**DOI:** 10.3389/fpsyg.2020.566886

**Published:** 2020-09-25

**Authors:** Claus-Christian Carbon

**Affiliations:** ^1^Department of General Psychology and Methodology, University of Bamberg, Bamberg, Germany; ^2^Research Group EPÆG (Ergonomics, Psychological Aesthetics, Gestalt), Bamberg, Germany

**Keywords:** emotion, face masks, accuracy, confusion, COVID-19, pandemic, mouth

## Abstract

Wearing face masks is one of the essential means to prevent the transmission of certain respiratory diseases such as coronavirus disease 2019 (COVID-19). Although acceptance of such masks is increasing in the Western hemisphere, many people feel that social interaction is affected by wearing a mask. In the present experiment, we tested the impact of face masks on the readability of emotions. The participants (*N* = 41, calculated by an *a priori* power test; random sample; healthy persons of different ages, 18–87 years) assessed the emotional expressions displayed by 12 different faces. Each face was randomly presented with six different expressions (*angry*, *disgusted*, *fearful*, *happy*, *neutral*, and *sad*) while being fully visible or partly covered by a face mask. Lower accuracy and lower confidence in one’s own assessment of the displayed emotions indicate that emotional reading was strongly irritated by the presence of a mask. We further detected specific confusion patterns, mostly pronounced in the case of misinterpreting disgusted faces as being angry plus assessing many other emotions (e.g., *happy*, *sad*, and *angry*) as neutral. We discuss compensatory actions that can keep social interaction effective (e.g., body language, gesture, and verbal communication), even when relevant visual information is crucially reduced.

## Introduction

Wearing face masks^1^ is recommended in many scenarios, mostly in clinical contexts, when infected by certain respiratory diseases or in times of epidemics where the risk of potential transmission through air passages has to be reduced (Jefferson et al., 2008). During the coronavirus disease 2019 (COVID-19) pandemic, most countries and health organizations like the WHO propagated wearing face masks by early 2020 as a key strategy to reduce the spread of the severe acute respiratory syndrome 2 (SARS 2) coronavirus.

Face masks not only have a direct positive medical impact in terms of preventing the virus from spreading to those who are most vulnerable (Wu and McGoogan, 2020); they also have positive societal effects as wearing masks allows for the relaxing of other preventive measures such as strict isolation and quarantining (Mniszewski et al., 2014). However, face masks also cover, per definition, a major part of the human face, which can crucially affect social interaction. Our faces provide the key information of personal identity; additional socially important information such as trustworthiness, attractiveness, age, and sex; information that supports the understanding of speech by enabling facial speech analysis, as well as fine-grained information that allows for reading the other’s emotional state *via* expression analysis (Bruce and Young, 1986). We can compensate for a lack of signal for all of these facets of face processing (Grüter and Carbon, 2010); for instance, even strong cases of congenital prosopagnosia – a cognitive dysfunction that impairs or even disables the ability to recognize persons by faces (therefore, often misleadingly called “face blindness”) – are mostly overlooked in society. Although congenital prosopagnosia shows a high prevalence rate of about 2.5% (Grüter et al., 2008), we rarely encounter a person who explicitly shows this inability in real-life – the reason for this is that many of the affected persons have developed coping strategies. For instance, they compensate for the impaired capability of reading facial identification cues by means of using different sources of information such as the characteristic gait or gesture, or by using information from other modalities, such as the characteristic voice pattern of a person. But even with successful compensation, the efficacy of processing is often reduced. This is also reflected in the confidence of one’s assessments. Actually, the affected persons are susceptible to losing a part of the multichannel-multisensory integration possibilities to crosscheck and validate their assessments. Some of these signals that faces provide are processed very fast (identity, Carbon, 2011; gender and attractiveness, Carbon et al., 2018; emotion, Willis and Todorov, 2006), although the validity of the final assessments is under great dispute (Russell, 1994; Rojahn et al., 2000).

With regard to expression analysis, different studies have showed that we are far from perfect in assessing the emotional state of our counterpart. This is especially the case when we just rely on pure facial information (Derntl et al., 2009) without knowing the context of a scene (Aviezer et al., 2008). Another factor that lowers our performance in correctly reading emotions from faces is the static view on faces without any information about the dynamic progression of the seen expression (Bassili, 1979; Blais et al., 2012, 2017). A partial occlusion of the face (Bassili, 1979), e.g., by sunglasses (Roberson et al., 2012) or by scarfs (Kret and de Gelder, 2012), is a further obstacle to accurately reading emotions from facial expressions (Bassili, 1979). Face masks or community masks, as the ones commonly worn during the COVID-19 pandemic to shield the mouth and the nose, cover about 60–70% of the area of the face that is relevant for emotional expression, and thus, emotion reading (e.g., ~65% in the case of the depicted persons in our face set – exact numbers are hard to tell; we can only rely on rough estimations as indicative face areas differ from person to person). Crucially, these masks cover an area of the face that is crucial for the effective nonverbal communication of emotional states. Although specific research on the impact of such face masks on emotional recognition is missing, there are some indications from research on the effect of different kinds of facial occlusions. An important source of data is the so-called “Bubbles”-paradigm that make use of a general technique developed by Gosselin and Schyns (2001). This technique allows for identifying the specific visual information that is most relevant to human categorization performance, for instance, information needed to express and read emotions. Of special relevance regarding the Bubbles technique are findings that specifically addressed the specific parts of faces that are most indicative for certain emotional expressions (e.g., Smith et al., 2005; Blais et al., 2012). Blais et al. (2012), for instance, revealed the paramount importance of processing the mouth region. With a clever combination of a Bubbles paradigm and dynamic face stimuli from video sequences of half a second length starting with neutral expression that naturally deployed into an expressive state ending with the apex of the expression, the authors even demonstrated that this dominance of the mouth region persisted nearly over the entire period of time. Other paradigms comprise the presentation of top vs. bottom halves of faces (Bassili, 1979) or the partial occlusion of target faces with ecological valid items such as a niqāb (Fischer et al., 2012), a shawl, or a cap (Kret and de Gelder, 2012) in order to test for differences in the participants’ emotion reading performance. These different paradigms operate with very different stimuli, and they were used with samples from different populations. In any case, the found effects are informative for the present study as specific emotions were primarily hard to read in faces with occlusions of the mouth area; for instance, happiness (for occlusions by a rectangular cardboard, see Bassili, 1979; for occlusions by a niqāb, see Fischer et al., 2012; Kret and de Gelder, 2012) or sadness (for occlusions by a rectangular cardboard, see Bassili, 1979; for occlusions by a niqāb, see Fischer et al., 2012; Kret and de Gelder, 2012), while anger, for instance, was affected much less and remained observable (for occlusions by a rectangular cardboard, see Bassili, 1979; for occlusions by a niqāb, see Fischer et al., 2012; Kret and de Gelder, 2012). Taken together, these studies provide excellent basic data on how strongly and selectively occlusions of the mouth area affect the recognition of facial emotion, but they did not specifically address how face masks impact the reading of different emotions. The manipulations realized in those paradigms are neither quantitatively nor qualitatively analogous to the actual practical use of face masks. By using face masks, we can also check whether they operate as a kind of psychological marker for disease, a deliberate disguise, or indicate some special status of the wearer; it is also possible that a face mask can signal a potentially dangerous situation by triggering anxiety-related associations – a marker operating in such a way could modulate the interpretation of the entire social situation and so also of the specific emotional expression. The results of the existing studies show some clear common ground, for instance, a relatively high consensus that covering the lower face parts, especially the mouth (Blais et al., 2012), yields reduced performance in assessing a happy emotional state (e.g., Kotsia et al., 2008; Eisenbarth and Alpers, 2011; Fischer et al., 2012). For other emotional states than happy faces, however, there are quite contradictory results to be found in the literature, e.g., for fear detection (in favor of higher relevance of the eyes, see Bombari et al., 2013; in favor of higher relevance of the mouth, see Kotsia et al., 2008). There is even evidence that a partial covering of the face might lead to *better* performance due to blocking out irrelevant or deceptive information in faces (Kret and de Gelder, 2012). Laypersons, for instance, were more accurate in detecting deception in persons who wore a niqāb than in persons who did not (Leach et al., 2016). Inconsistent results such as angry faces attracting more attention to the eyes than the mouth (Eisenbarth and Alpers, 2011) while the occlusion of the mouth resulted in lower accuracy of detecting anger (Kotsia et al., 2008) have to be interpreted with caution as we do not know the causal or temporal interdependence of such processes. Specific types of occlusions might interfere with different emotions: for example, the mouth seems important for the detection of happiness and fear, but the eyes are more relevant for anger, fear, and sadness (Bombari et al., 2013).

The present study specifically tested how a common face mask, which, for instance, dominates social scenes during the COVID-19 pandemic, changes the efficacy of emotion reading expressions displayed by different faces. Besides recognition sensitivity, in order to understand everyday life problems in effectively communicating when wearing face masks, we were particularly interested in the confusion of certain emotions with other emotional states due to an increase in signal ambiguity.

## Experimental Study

### Methods

#### Participants

The needed sample size of *N* = 36 was calculated *a priori via* power analysis (Faul et al., 2007) targeting a repeated measures analysis of variance (ANOVA) with six groups (emotions) and two measurements (mask vs. no mask) and the ability to detect a medium effect size of *f* = 0.25 (Cohen, 1988), given an *α* = 0.05 and a test power (1-β) = 0.80. From our entire set of data from 41 participants [*M*_age_ = 26.7 years (18–87 years), *N*_female_ = 30], we could use all data sets as all participants reached the pre-defined criterion of showing at least a performance of correctly identifying emotional states in 50% of the cases where faces were presented without masks (actually, the performance was much higher, see results). This slightly higher actual than needed number of participants resulted in an achieved *post hoc* test power of 0.88.

#### Material

**Figure 1:**
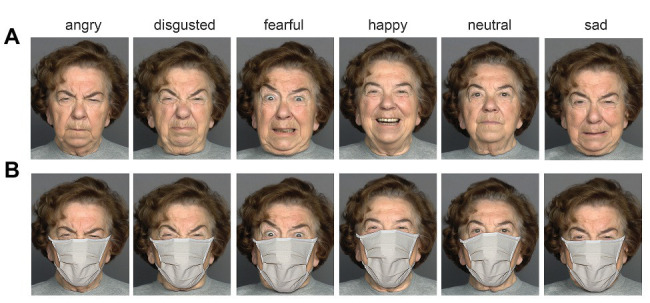
A person showing six different emotions without a mask **(A)** and wearing a mask **(B).** Original material from top row stems from MPI FACES database (Ebner et al., 2010).

All face stimuli were obtained from the MPI FACES database (Ebner et al., 2010) by a study-specific contract effective by 27 April 2020. As base faces on which we later applied face masks, we used frontal photos of 12 Caucasians (six females and six males) who belonged to three different face age groups (*young*, *medium* = middle-aged, and *elderly*), yielding two persons per *face sex* × *face age group* cell. For each person, six different pictures were used that showed the emotional states *angry*, *disgusted*, *fearful*, *happy*, *neutral*, and *sad*. For the application of face masks to all of these 72 original pictures, we photographed a typical homemade (beige) community mask. The image of the mask was cut out *via* Photoshop and individually applied to the different face versions. Realistic shadows were added to create maximally realistic and plastic pictures of persons wearing a face mask (Figure 1).

In sum, we obtained 2 (face sex) × 3 (face age group) × 2 (individuals) × 6 (emotions) × 2 (no face mask vs. face mask) = 144 face stimuli.

#### Procedure

The experiment which ran on the SoSciSurvey online platform was conducted between 15 May (10:01 local time) and 18 May (19:45 local time) during the COVID-19 pandemic when general legal obligations to wear masks in Germany were already in action. Prior to the experimental session, written informed consent was obtained from each participant. All data were collected anonymously. Each participant was exposed to the complete set of stimuli one after another, with the order of stimuli being randomized across participants. Participants were asked to spontaneously assess the depicted person’s emotional state from a list of six emotions reflecting the same compilation of emotions shown by the different versions of the faces (*angry*, *disgusted*, *fearful*, *happy*, *neutral*, and *sad*). Personal confidence for each assessment had to be indicated on a scale from 1 (*very unconfident*) to 7 (*very confident*). There was no time limit for giving a response. The general study design (psychophysical testing) was given ethical approval by the local ethics committee of the University of Bamberg. The entire procedure lasted approximately 20–25 min.

## Results

Data were submitted to further data processing executed by R 4.0.0 (R Core Team, 2014), with linear mixed models (LMMs) being analyzed *via* toolbox *lmer* (Kuznetsova et al., 2017). The entire anonymized data set is available at the Open Science Framework.^2^

**Figure 2:**
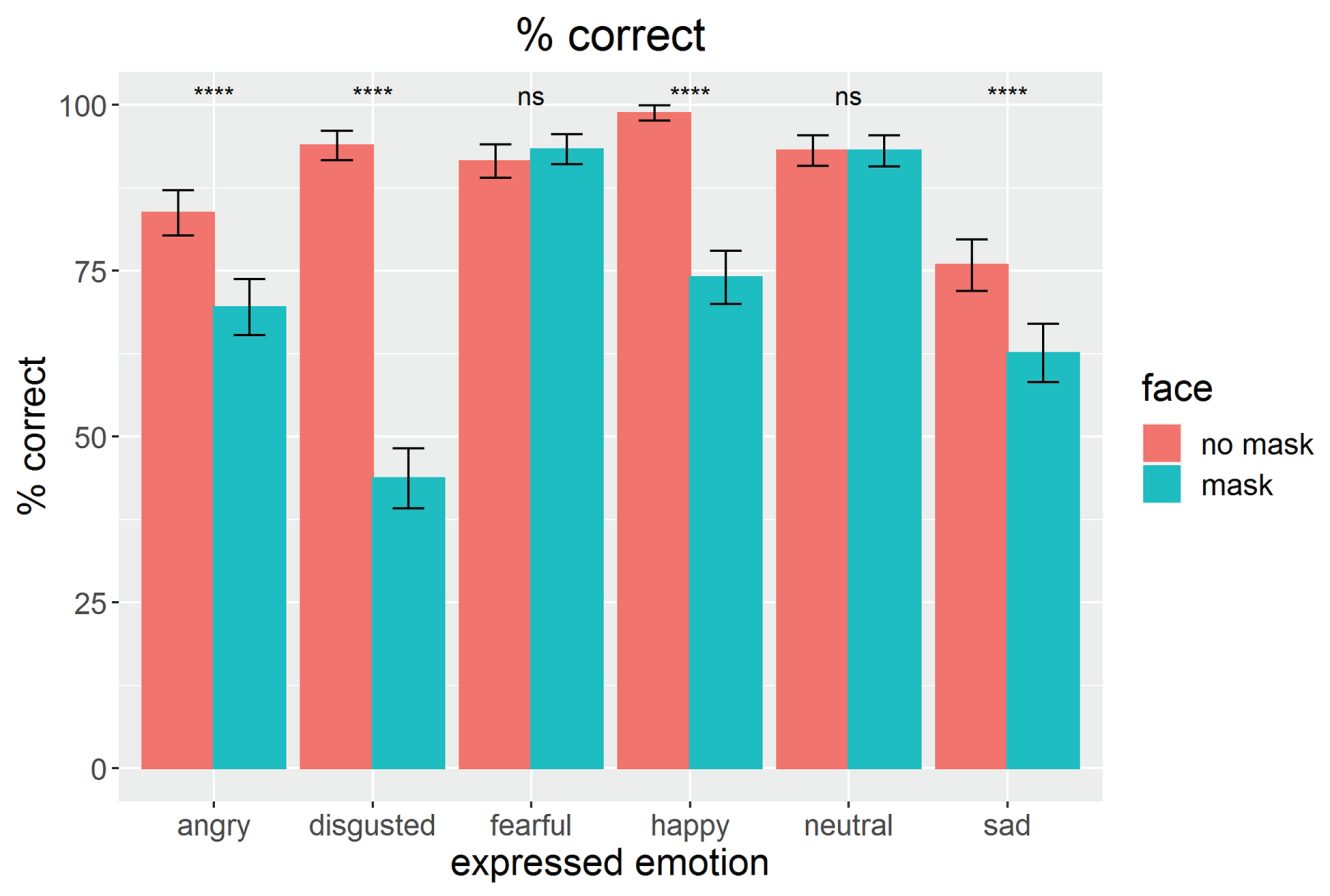
Mean percentage of correct assessment of the emotional states for faces with masks (blue) or without masks (red) on the face. Error bars indicate confidence intervals CI-95% based on adjusted values for taking within-subject variances into account (Morey, 2008). Asterisks indicate statistical differences between conditions of wearing and non-wearing on the basis of paired *t*-tests: ^****^p < 0.0001; ns, not significant.

Overall performance for correctly identifying facial emotions in faces without masks was quite remarkable, *M* = 89.5% (chance rate = 16.7%) with no participant performing below an overall rate of 76.4%; this high recognition rate outperforms the accuracy of assigning emotional states to faces documented by many other studies (for anger and disgust 56.9 and 58.9%, respectively, see Aviezer et al., 2008; 73.2, 73.7, 63.2, and 72.2%, for sadness, anger, disgust, and fear, respectively, see Derntl et al., 2009). As shown by the mean data for each emotional state (Figure 2), presenting a mask on faces showed a clear performance drop in reading emotions in faces. With the exception of fearful and neutral faces, for which ceiling performance effects were observed, all emotional states were harder to read in faces with masks.

We tested the effect of wearing masks on the performance of emotional reading in faces by means of LMMs with *face mask* (face with a mask vs. without a mask) as a fixed factor against a base model (model #0) which only contained the participants and base stimuli as random intercepts and *face emotion* as fixed slopes – *FS* (fixed factors). We furthermore tested, in a successive way, the effect of the sex and age group of the face stimuli by adding these factors as *FS* – including all possible interactions of all fixed factors. *p*-values were obtained by likelihood ratio tests of the subsequent models against the respective one-step less complex model. The coefficient of determination for each model was calculated via a likelihood-ratio test utilizing the toolbox *MuMIn* (Barton, 2019). See Table 1 for detailed results.

**Table 1:** Linear mixed effect analysis of different models in comparison to a simple base model (model #0), separated by the two tested dependent variables %correct (percentage of correct emotion classifications) and confidence (for correct emotion classifications).

Dependent variable/tested model	df	AIC	logLik	*R*^2^	*p*(*χ*^2^)
**%correct**					
#0: base (random intercepts)	9	59,598	−29,790	0.090	
#1: +FS face mask	15	58,945	−29,458	0.187	<0.0001
#2: +FS face sex	27	58,850	−29,398	0.203	<0.0001
**#3: +FS face age group**	**75**	**58,465**	**−29,157**	**0.266**	**<0.0001**
**Confidence**					
#0: base (random intercepts)	9	16,174	−8,078	0.161	
#1: +FS face mask	15	15,171	−7,571	0.321	<0.0001
#2: +FS face sex	16	15,173	−7,570	0.321	0.604 *ns*
**#3: +FS face age group**	**75**	**15,021**	**−7,436**	**0.358**	**<0.0001**

The best fitting model, while being parsimonious, is indicated by bold face. FS, fixed slopes (fixed factors); RS, random slopes (random factors); df, degrees of freedom; *R*^2^, coefficient of determination, based on the likelihood-ratio test; *p*(*χ*^2^), probability of accepting a significant effect despite a non-existent difference regarding the more complex vs. the one-step less complex model.

Linear mixed effect analysis revealed that both dependent variables were impacted by the factor *face mask*. Furthermore, *face age group* played a role in explaining the variance of both dependent variables (reading the emotional status of elderly faces was more difficult than reading it from middle-aged or young faces; this effect was pronounced when faces were shown with masks) – for *face sex*, in contrast, we only found an effect for the accuracy of emotion reading.

**Figure 3:**
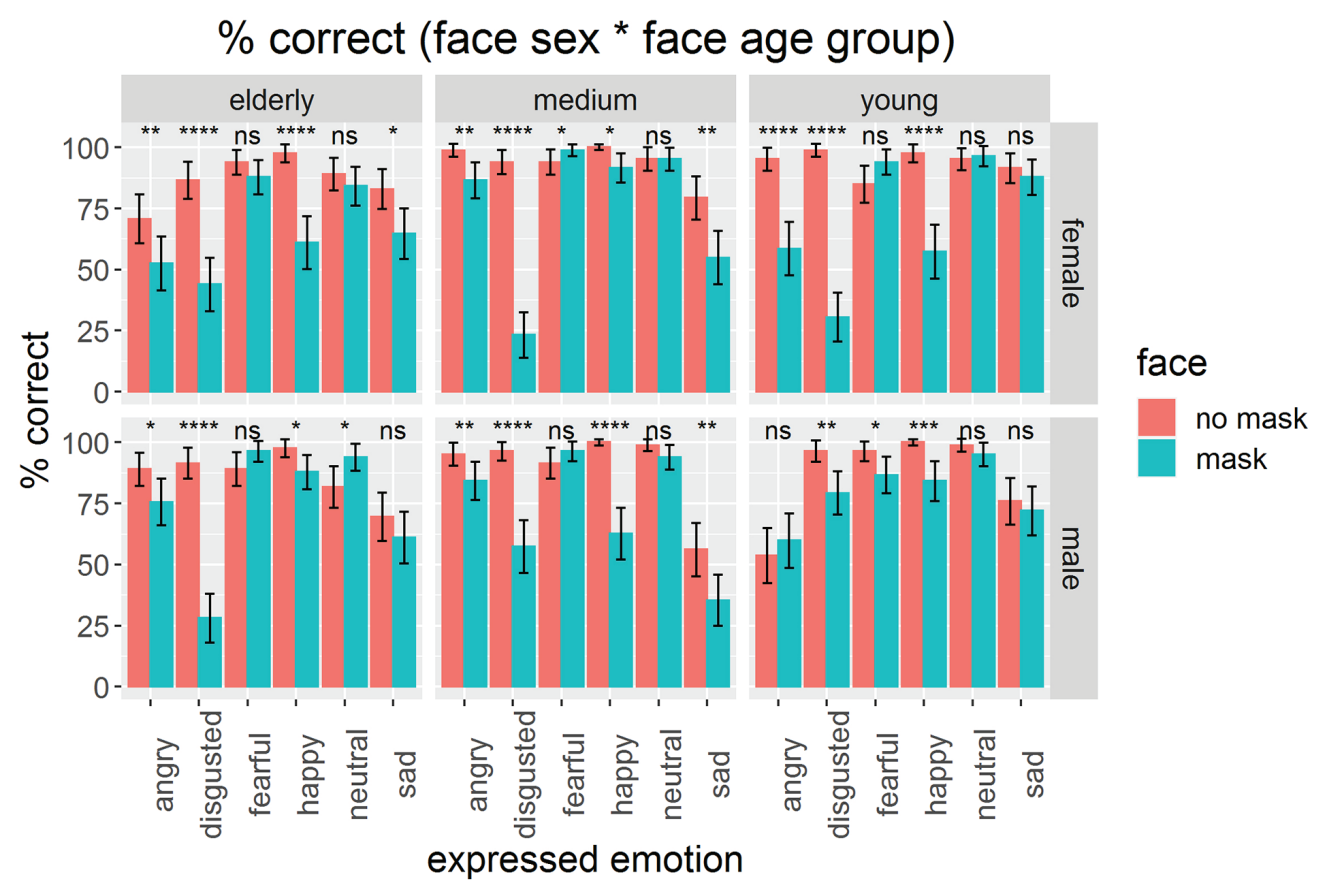
Mean percentage of correctly assessing the emotional states with masks (blue) or without masks (red) on the face, split by face sex and face age group. Error bars indicate confidence intervals CI-95% based on adjusted values for taking within-subject variances into account (Morey, 2008). Asterisks indicate statistical differences between conditions of wearing and non-wearing on basis of paired *t*-tests: ^*^p < 0.05, ^**^p < 0.01, ^***^p < 0.001, ^****^p < 0.0001; ns, not significant.

As *face sex* as well as *face age group* were effective in predicting the correctness of reading the emotional state of faces, Figure 3 shows the differentiated data for the three-way interactive effect with *face mask*. Lower performance in assessing emotions in masked faces was found for most emotions and sex and age groups.

Based on the finally selected models with *face mask*, *face sex*, and *face age group* being included in terms of fixed slopes and their interactions, we obtained several effects of small, medium, and large size (Table 2). Most importantly, regarding the major question of the study, *face mask* had a medium-sized effect on the performance of assessing the emotional state of a face and a large-sized effect on the confidence of one’s own assessment (for correct emotion classifications).

**Table 2:** Statistics of all involved fixed effects terms of the linear mixed effect analysis for the final models (model #3), separated by the two tested dependent variables %correct (percentage of correct emotion classifications) and confidence (for correct emotion classifications).

			%correct	Confidence
	Term	*k*(par)	Cohen’s *f*	Cohen’s *f*
1	Emotion	5	0.304 *medium*	0.263 *medium*
2	Mask	1	0.253 *medium*	0.458 *large*
3	Sex	1	0.002	0.015
4	Age	2	0.017	0.045
5	Emotion:mask	5	0.263 *medium*	0.204 *small*
6	Emotion:sex	5	0.122 *small*	0.060
7	Mask:sex	1	0.062	0.002
8	Emotion:age	10	0.193 *small*	0.159 *small*
9	Mask:age	2	0.019	0.045
10	Sex:age	2	0.012	0.037
11	Emotion:mask:sex	5	0.059	0.055
12	Emotion:mask:age	10	0.061	0.054
13	Emotion:sex:age	10	0.150 *small*	0.095
14	Mask:sex:age	2	0.047	0.032
15	Emotion:mask:sex:age	10	0.137 *small*	0.096

*k*(par), number of parameters; Cohen’s *f*, effect size including qualification as small, medium, or large according to Cohen (1988), smaller effects are not further qualified. Abbreviated notations for the terms were used to save space, emotion, face emotion; mask, face mask; sex, face sex; age, face age group.

**Figure 4:**
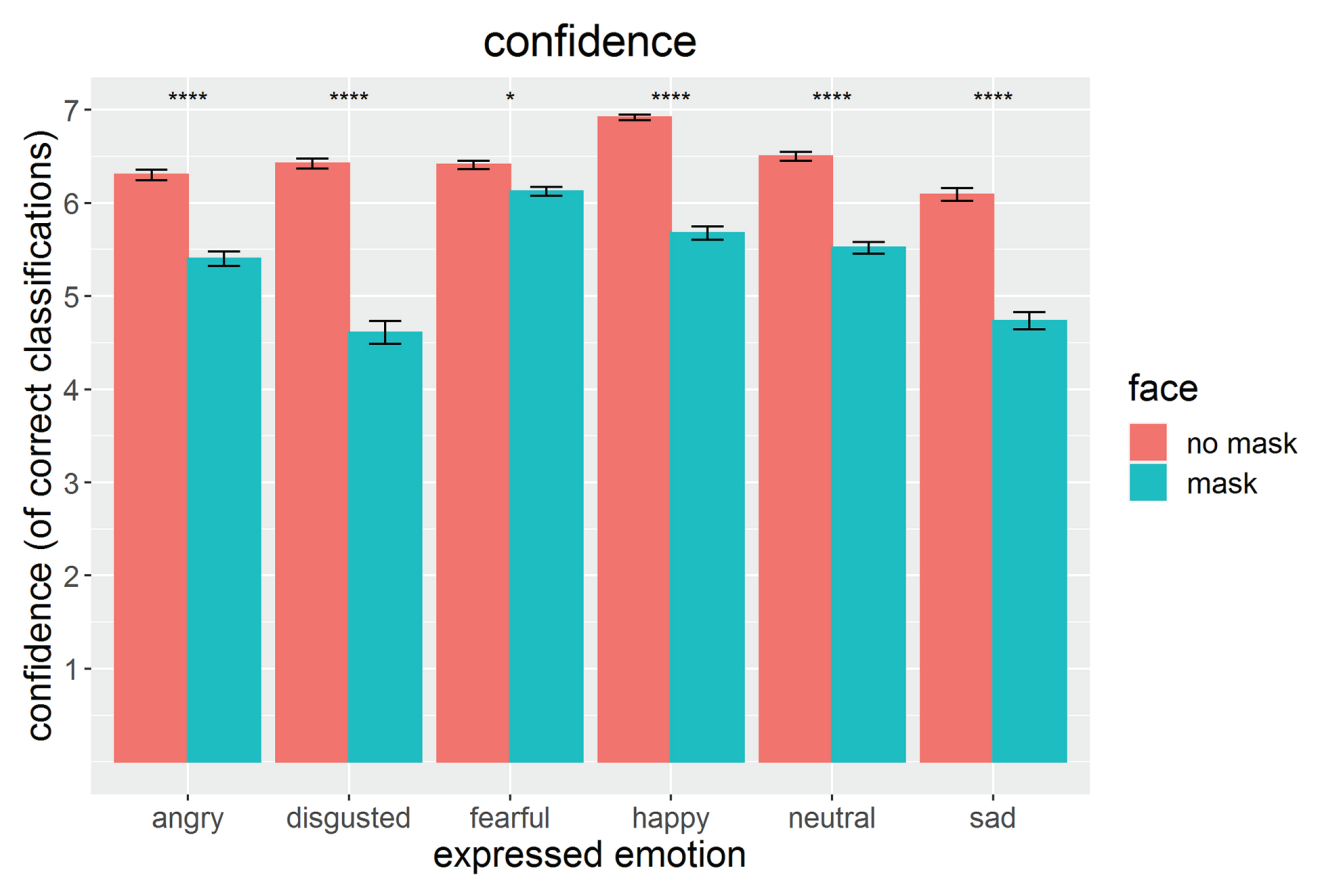
Mean confidence of assessing the emotional states (for correct classifications) with masks (blue) or without masks (red) on the face. Error bars indicate confidence intervals CI-95% based on adjusted values for taking within-subject variances into account (Morey, 2008). Asterisks indicate statistical differences between conditions of wearing and non-wearing on basis of paired *t*-tests: ^*^p < 0.05, ^****^p < 0.0001; ns, not significant.

As shown in Figure 4, the confidence data showed a similar but not identical results pattern compared to the percentage of correct assessment data in Figure 2. Interestingly, confidence data reflected the impact of a face mask emotion reading even more clearly. For confidence ratings, fearful and neutral faces were also impacted, probably due to a lack of ceiling effects.

A drop in performance in reading the emotional states of faces with masks can somehow be expected as being much harder when most visual information of the lower half of the face is blocked out. To understand how the lack of information is dealt with, it is important to look at the specific confusion of individual emotional states – when and in which way are emotions misinterpreted when face masks are worn?

**Figure 5:**
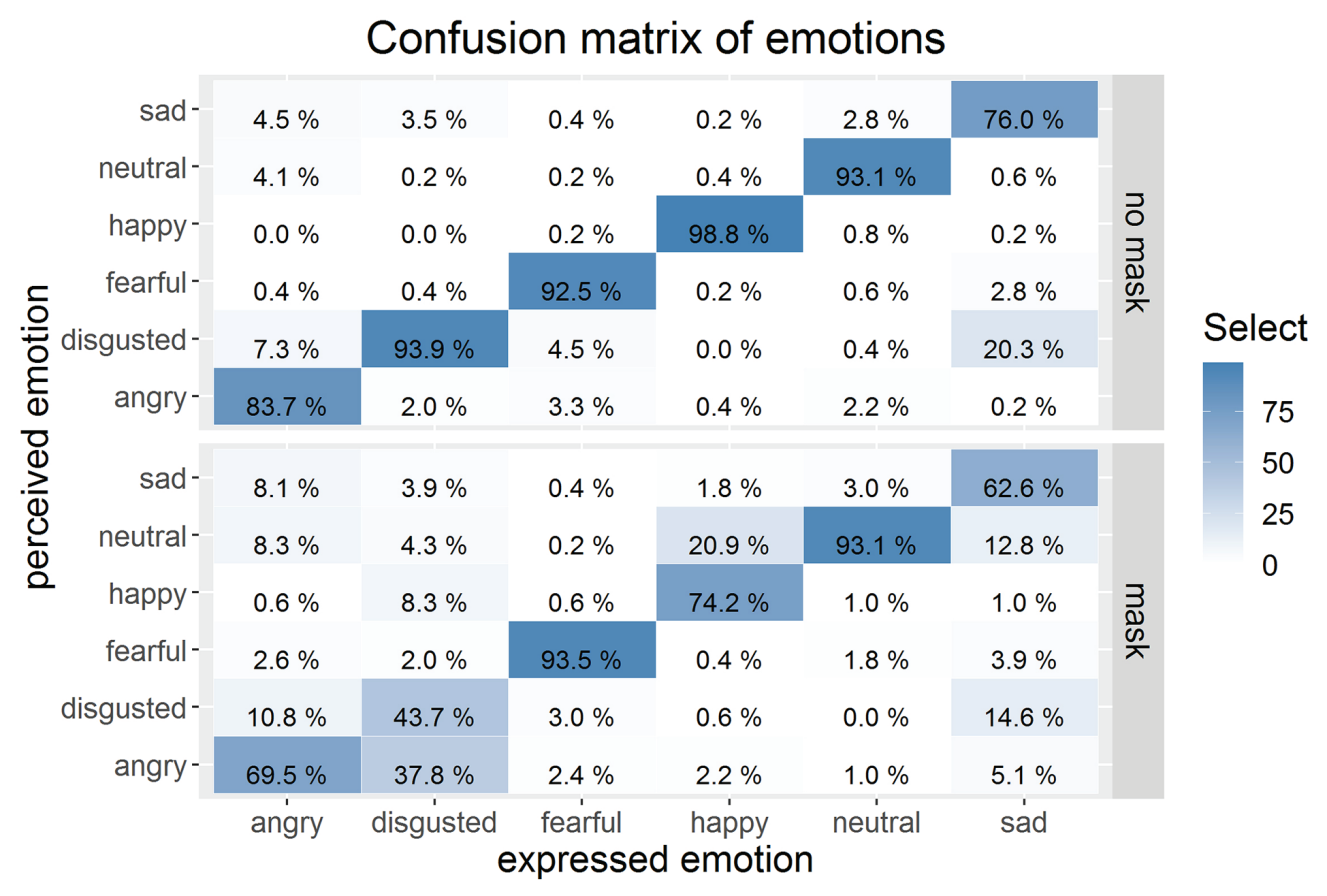
Confusion matrix of expressed and perceived emotions. Top matrix: faces without masks, bottom matrix: faces with a mask. Percentages compile up to 100% for each expressed emotion. The deeper blue the cell, the higher the score of this cell.

In order to learn about these misinterpretations, we generated confusion matrices for the viewing conditions for faces without masks and with masks (see Figure 5). When faces were shown without masks, the accuracy was much higher, as is indicated by clear matches between expressed and perceived emotions. With the exception of the emotional state *sad*, accuracy was above 83%, but *sad*, in particular, was often confused with *disgusted* (20.3% of the cases). As soon as we applied masks to the faces, this overall very high performance broke down dramatically and characteristic confusions became apparent. For instance, all emotional states with the exception of *fearful* were repeatedly confused with a neutral state. *Sad* was often confused with *disgusted* and *neutral*, and *angry* was confused with *disgusted*, *neutral*, and *sad*. Most drastically was the misinterpretation of *disgusted* as *angry*, which showed up in nearly 38% of the cases, although such a confusion did only happen in 2% of the cases where no face mask was used. In previous studies, it was shown that, in particular, the recognition of the emotional states *happy* and *sad*, and to a smaller degree *angry*, rely strongly on the processing of the lower facial part, especially the mouth area (Bassili, 1979; Fischer et al., 2012; Kret and de Gelder, 2012). And, exactly these emotional states were hard to decipher and easily confounded when a mask was applied to the target face.

The statistics on the confusion of emotions show clearly how ambiguous an emotional state becomes when an ordinary face mask is worn.

## Discussion

Wearing face masks, even very simple homemade models, is an important measure to effectively decrease the chance of transmitting respiratory diseases (van der Sande et al., 2008), as is also suggested by the analysis of past pandemics such as the *1918 flu pandemic* caused by the H1N1 influenza (Bootsma and Ferguson, 2007). People in countries where face masks have not been widely used in the past may still be ambivalent about wearing them. Acceptance of wearing a mask is low when surrounded by too many non-wearers – people start to feel “strange” (Carbon, 2020); additionally, there are obvious handling problems and ergonomic issues including changed airflow characteristics which do not support the wearing of masks. Yet, the usage of masks is becoming an everyday practice all over the world, including Europe where the wearing of masks was uncommon before the COVID-19 pandemic.

In the present experiment, we tested the impact of face masks on emotion reading, which may have important implications for everyday social interaction. We confronted participants with faces showing six different emotions (*angry*, *disgusted*, *fearful*, *happy*, *neutral*, and *sad*). The results indicate that emotion recognition was strongly reduced with the exception of fearful and neutral faces, which is compatible with parts of the literature employing different types of occlusion, for instance, by rigidly covering the mouth area with cardboard (Bassili, 1979), using the Bubbles technique (Blais et al., 2012), or, much closer to the present study, with ecologically valid paraphernalia such as a niqāb (Fischer et al., 2012), a shawl or a cap (Kret and de Gelder, 2012). For fearful faces, as shown before in the literature (but see Kret and de Gelder, 2012; Bombari et al., 2013; Wegrzyn et al., 2017), the eye region, which was not occluded by the mask, provides most of the emotional information indicative for this emotional state. For neutral faces, the results have to be interpreted in a completely different and cautious way: although performance for recognizing a neutral state was not directly decreased, many emotional states such as *happy*, *sad*, and *angry* were misinterpreted as *neutral*, so the genuine emotional state was not perceived anymore. Other emotions such as *disgusted* were confused with *angry*, and this qualitative misinterpretation – which is quite impactful (a person who does feel aversion to a very specific thing in a certain situation and who expresses this spontaneously might be interpreted as an angry and potentially aggressive person) – was found in more than one third of all assessments of disgusted faces wearing a mask.

To further qualify these effects, we have to make it clear that the face stimuli originated from a scientific database, which is aiming to show emotions with maximal clarity and in a very pronounced fashion. These requirements were nearly perfectly achieved when we look at the very high performance data for the original faces without masks. There was hardly any confusion of different emotional states (with the exception of *sad* faces which already showed substantial confounding with *disgusted* at a level of one-fifth of the cases). Such a high performance is hardly achievable in everyday life when faces are inspected that show much lower degrees of emotional expression. Furthermore, in an everyday life scene, we will typically show lower levels of attention and will invest less time in inspecting the face of a counterpart. This means that in natural contexts, the impact of face masks on reading emotions could even be stronger. It could further be intensified with increased age: as the results of some empirical studies indicate, older adults have more difficulty recognizing some of the basic emotions (e.g., disgust, happiness, and fear), and even intense problems in recognizing other basic emotions such as anger and sadness (Ruffman et al., 2008). On the other hand, we also have to make clear that the data presented here are based on the processing of graphically manipulated stimuli, not on faces wearing masks in a real world scenario. We opted for such a solution because if we photograph the same person wearing a mask vs. wearing no mask under the condition of six different emotions, the change in emotional expression is no longer controllable. Experimental designs are always in the difficult situation of finding an optimal balance between internal and external or even ecological validity. So, we took great care to present realistic and highly plausible stimuli which were graphically post-processed by adding shadows and adjusting them to the sizes and directions of the heads. Having taken this path, we cannot exclude that people in real-world settings will adjust to the situation of wearing masks and compensate the lack of expression options by amplifying their expressions. Everyday life experience contradicts this idea as people frequently report such confusions of emotions and complain about the lack of confidence in others’ emotional states, which we have also documented in the present paper.

Face masks may complicate social interaction as they disturb emotion reading from facial expression. This should, however, not be taken as a reason or an excuse for not wearing masks in situations where they are of medical use. We should not forget that humans possess a variety of means to interpret another’s state of mind, including another’s emotional states. Facial expressions are not our one and only source of information; we can also take recourse to body posture and body language to infer the emotional states of our counterpart. The voice characteristic adds indications from another modality (Golan et al., 2006), and the bodily context (Aviezer et al., 2008), the head orientation (Sauer et al., 2014, but also just inspect Figure 1 with a clear sign of specific head orientations as a by-product of emotional expression), and, of course, also the social context (Mondloch, 2012) will provide further information. Direct verbal communication even helps to understand the very fine-tuned state of a mind. We have options, and it is essential to make use of them not only when being the receiver of socially relevant information but also when being the sender. And, we should use and optimize those options which we can best play and which suit us best; this not only applies for times and situations where we cover parts of our face due to health or cultural reasons but extends to cases where the ability to express emotions is affected (e.g., due to neurological diseases, Jin et al., 2017; Lee et al., 2019): some people might have only a very limited repertoire of gestures and other body-oriented expression abilities but they might be good verbal communicators. Emphasizing alternative and additional communicative channels (see Aviezer et al., 2008), we can provide sufficient information to keep social interaction going in a different, yet, effective way.

## Data Availability Statement

The datasets presented in this study can be found in online repositories. The names of the repository/repositories and accession number can be found at: https://osf.io/ka3s6/.

## Ethics Statement

This study was approved in terms of the general study design (psychophysical testing) and was given ethical approval by the local ethics committee of the University of Bamberg (protocol 2017-08-18). The patients/participants provided their written informed consent to participate in this study.

## Author Contributions

C-CC had the initial idea, prepared the material, conducted the study, analyzed the data, and wrote the paper.

### Conflict of Interest

The author declares that the research was conducted in the absence of any commercial or financial relationships that could be construed as a potential conflict of interest.

## Footnotes

^1^Face masks show a great variety of forms and technologies; within the present paper, we will focus on masks that look like simple surgical masks and that people can fabricate themselves, so called *community masks*.

^2^https://osf.io/ka3s6/

## References

[ref1] Aviezer H. Hassin R. R. Ryan J. Grady C. Susskind J. Anderson A.. (2008). Angry, disgusted, or afraid? Studies on the malleability of emotion perception. Psychol. Sci. 19, 724–732. 10.1111/j.1467-9280.2008.02148.x, PMID: 18727789

[ref42] Barton K. (2019). MuMIn: Multi-Model Inference. R package version 1.43.6. Available at: https://CRAN.R-project.org/package=MuMIn

[ref2] Bassili J. N. (1979). Emotion recognition: the role of facial movement and the relative importance of upper and lower areas of the face. J. Pers. Soc. Psychol. 37, 2049–2058. 10.1037/0022-3514.37.11.2049, PMID: 521902

[ref3] Blais C. Fiset D. Roy C. Saumure Régimbald C. Gosselin F. (2017). Eye fixation patterns for categorizing static and dynamic facial expressions. Emotion 17, 1107–1119. 10.1037/emo0000283, PMID: 28368152

[ref4] Blais C. Roy C. Fiset D. Arguin M. Gosselin F. (2012). The eyes are not the window to basic emotions. Neuropsychologia 50, 2830–2838. 10.1016/j.neuropsychologia.2012.08.010, PMID: 22974675

[ref5] Bombari D. Schmid P. C. Schmid Mast M. Birri S. Mast F. W. Lobmaier J. S. (2013). Emotion recognition: the role of featural and configural face information. Q. J. Exp. Psychol. 66, 2426–2442. 10.1080/17470218.2013.789065, PMID: 23679155

[ref6] Bootsma M. C. J. Ferguson N. M. (2007). The effect of public health measures on the 1918 influenza pandemic in US cities. Proc. Natl. Acad. Sci. U. S. A. 104, 7588–7593. 10.1073/pnas.0611071104, PMID: 17416677 PMC1849868

[ref7] Bruce V. Young A. (1986). Understanding face recognition. Br. J. Psychol. 77, 305–327. 10.1111/j.2044-8295.1986.tb02199.x, PMID: 3756376

[ref8] Carbon C. C. (2011). The first 100 milliseconds of a face: on the microgenesis of early face processing. Percept. Mot. Skills 113, 859–874. 10.2466/07.17.22.Pms.113.6.859-874, PMID: 22403930

[ref9] Carbon C. C. (2020). The psychology of wearing face masks in times of the COVID-19 pandemic. Available at SSRN: https://ssrn.com/abstract=3584834 (Accessed August 30, 2020).

[ref10] Carbon C. C. Faerber S. J. Augustin M. D. Mitterer B. Hutzler F. (2018). First gender, then attractiveness: indications of gender-specific attractiveness processing via ERP onsets. Neurosci. Lett. 686, 186–192. 10.1016/j.neulet.2018.09.009, PMID: 30217503

[ref11] Cohen J. (1988). Statistical power analysis for the behavioral sciences. 2nd Edn. New York: Lawrence Erlbaum Associates.

[ref12] Derntl B. Seidel E. M. Kainz E. Carbon C. C. (2009). Recognition of emotional expressions is affected by inversion and presentation time. Perception 38, 1849–1862. 10.1068/P6448, PMID: 20192133

[ref13] Ebner N. C. Riediger M. Lindenberger U. (2010). FACES-A database of facial expressions in young, middle-aged, and older women and men: development and validation. Behav. Res. Methods 42, 351–362. 10.3758/brm.42.1.351, PMID: 20160315

[ref14] Eisenbarth H. Alpers G. W. (2011). Happy mouth and sad eyes: scanning emotional facial expressions. Emotion 11, 860–865. 10.1037/a0022758, PMID: 21859204

[ref15] Faul F. Erdfelder E. Lang A. -G. Buchner A. (2007). G*Power 3: a flexible statistical power analysis program for the social, behavioral, and biomedical sciences. Behav. Res. Methods 39, 175–191. 10.3758/BF03193146, PMID: 17695343

[ref16] Fischer A. H. Gillebaart M. Rotteveel M. Becker D. Vliek M. (2012). Veiled emotions: the effect of covered faces on emotion perception and attitudes. Soc. Psychol. Pers. Sci. 3, 266–273. 10.1177/1948550611418534

[ref17] Golan O. Baron-Cohen S. Hill J. (2006). The Cambridge mindreading (CAM) face-voice battery: testing complex emotion recognition in adults with and without Asperger syndrome. J. Autism Dev. Disord. 36, 169–183. 10.1007/s10803-005-0057-y, PMID: 16477515

[ref18] Gosselin F. Schyns P. G. (2001). Bubbles: a technique to reveal the use of information in recognition tasks. Vis. Res. 41, 2261–2271. 10.1016/S0042-6989(01)00097-9, PMID: 11448718

[ref19] Grüter T. Carbon C. C. (2010). Escaping attention. Some cognitive disorders can be overlooked. Science 328, 435–436. 10.1126/science.1190432 20413479

[ref20] Grüter T. Grüter M. Carbon C. C. (2008). Neural and genetic foundations of face recognition and prosopagnosia. J. Neuropsychol. 2, 79–97. 10.1348/174866407X231001, PMID: 19334306

[ref21] Jefferson T. Foxlee R. Del Mar C. Dooley L. Ferroni E. Hewak B.. (2008). Physical interventions to interrupt or reduce the spread of respiratory viruses: systematic review. BMJ 336, 77–80. 10.1136/bmj.39393.510347.BE, PMID: 18042961 PMC2190272

[ref22] Jin Y. Z. Mao Z. Q. Ling Z. P. Xu X. Zhang Z. Y. Yu X. G. (2017). Altered emotional recognition and expression in patients with Parkinson’s disease. Neuropsychiatr. Dis. Treat. 13, 2891–2902. 10.2147/Ndt.S149227, PMID: 29225467 PMC5708195

[ref23] Kotsia I. Buciu I. Pitas I. (2008). An analysis of facial expression recognition under partial facial image occlusion. Image Vis. Comput. 26, 1052–1067. 10.1016/j.imavis.2007.11.004

[ref24] Kret M. E. de Gelder B. (2012). Islamic headdress influences how emotion is recognized from the eyes. Front. Psychol. 3:110. 10.3389/fpsyg.2012.00110, PMID: 22557983 PMC3322610

[ref25] Kuznetsova A. Brockhoff P. B. Rune H. B. Christensen A. P. (2017). {lmerTest} package: tests in linear mixed effects models. J. Stat. Softw. 82, 1–26. 10.18637/jss.v082.i13

[ref26] Leach A. -M. Ammar N. England D. N. Remigio L. M. Kleinberg B. Verschuere B. J. (2016). Less is more? Detecting lies in veiled witnesses. Law Hum. Behav. 40, 401–410. 10.1037/lhb0000189, PMID: 27348716

[ref27] Lee K. H. Lee J. Y. Boltz M. McConnell E. S. (2019). Emotional expression of persons with dementia: an integrative review with implications for evidence-based practice. Worldviews Evid. Based Nurs. 16, 344–351. 10.1111/wvn.12395, PMID: 31397542

[ref28] Mniszewski S. M. Del Valle S. Y. Priedhorsky R. Hyman J. M. Hickman K. S. (2014). “Understanding the impact of face mask usage through epidemic simulation of large social networks” in Theories and simulations of complex social systems. *Vol. 52* eds. Mago V. K. Dabbaghian V. (Heidelberg, Berlin: Springer), 97–115.

[ref29] Mondloch C. J. (2012). Sad or fearful? The influence of body posture on adults’ and children’s perception of facial displays of emotion. J. Exp. Child Psychol. 111, 180–196. 10.1016/j.jecp.2011.08.003, PMID: 21939983

[ref30] Morey R. D. (2008). Confidence intervals from normalized data: a correction to Cousineau (2005). Tutor. Quant. Methods Psychol. 4, 61–64. 10.20982/tqmp.04.2.p061

[ref31] R Core Team (2014). R: a language and environment for statistical computing. Available at: http://www.R-project.org/ (Accessed August 30, 2020).

[ref32] Roberson D. Kikutani M. Doge P. Whitaker L. Majid A. (2012). Shades of emotion: what the addition of sunglasses or masks to faces reveals about the development of facial expression processing. Cognition 125, 195–206. 10.1016/j.cognition.2012.06.018, PMID: 22892280

[ref33] Rojahn J. Gerhards F. Matlock S. T. Kroeger T. L. (2000). Reliability and validity studies of the facial discrimination task for emotion research. Psychiatry Res. 95, 169–181. 10.1016/s0165-1781(00)00169-4, PMID: 10963802

[ref34] Ruffman T. Henry J. D. Livingstone V. Phillips L. H. (2008). A meta-analytic review of emotion recognition and aging: implications for neuropsychological models of aging. Neurosci. Biobehav. Rev. 32, 863–881. 10.1016/j.neubiorev.2008.01.001, PMID: 18276008

[ref35] Russell J. A. (1994). Is there universal recognition of emotion from facial expression: a review of the cross-cultural studies. Psychol. Bull. 115, 102–141. 10.1037/0033-2909.115.1.102, PMID: 8202574

[ref36] Sauer A. Mothes-Lasch M. Miltner W. H. R. Straube T. (2014). Effects of gaze direction, head orientation and valence of facial expression on amygdala activity. Soc. Cogn. Affect. Neurosci. 9, 1246–1252. 10.1093/scan/nst100, PMID: 23946006 PMC4127025

[ref37] Smith M. L. Cottrell G. W. Gosselin F. Schyns P. G. (2005). Transmitting and decoding facial expressions. Psychol. Sci. 16, 184–189. 10.1111/j.0956-7976.2005.00801.x, PMID: 15733197

[ref38] van der Sande M. Teunis P. Sabel R. (2008). Professional and home-made face masks reduce exposure to respiratory infections among the general population. PLoS One 3:e2618. 10.1371/journal.pone.0002618, PMID: 18612429 PMC2440799

[ref39] Wegrzyn M. Vogt M. Kireclioglu B. Schneider J. Kissler J. (2017). Mapping the emotional face. How individual face parts contribute to successful emotion recognition. PLoS One 12:e0177239. 10.1371/journal.pone.0177239, PMID: 28493921 PMC5426715

[ref40] Willis J. Todorov A. (2006). First impressions: making up your mind after a 100-ms exposure to a face. Psychol. Sci. 17, 592–598. 10.1111/j.1467-9280.2006.01750.x, PMID: 16866745

[ref41] Wu Z. McGoogan J. M. (2020). Characteristics of and important lessons from the coronavirus disease 2019 (COVID-19) outbreak in China: summary of a report of 72314 cases from the Chinese Center for Disease Control and Prevention. JAMA 323, 1239–1242. 10.1001/jama.2020.2648, PMID: 32091533

